# Community benefits of mass distribution of three types of dual-active-ingredient long-lasting insecticidal nets against malaria prevalence in Tanzania: evidence from a 3-year cluster-randomized controlled trial

**DOI:** 10.1186/s12889-025-21586-x

**Published:** 2025-01-28

**Authors:** Eliud Andrea Lukole, Jackie Cook, Jacklin F Mosha, Nancy S Matowo, Manisha A Kulkarni, Elizabeth Mallya, Tatu Aziz, Jacklin Martin, Mark Rowland, Immo Kleinschmidt, Alphaxard Manjurano, Franklin W Mosha, Natacha Protopopoff

**Affiliations:** 1 Department of Parasitology, Mwanza Medical Research Centre, National Institute for Medical Research, Mwanza, Tanzania; 2 Department of Disease Control, London School of Hygiene and Tropical Medicine, London, UK; 3 MRC International Statistics and Epidemiology Group, Department of Infectious Disease Epidemiology, London School of Hygiene and Tropical Medicine, London, UK; 4 School of Epidemiology and Public Health, University of Ottawa, Ottawa, ON Canada; 5 Kilimanjaro Christian Medical University College, Moshi, Tanzania; 6 Wits Research Institute for Malaria, School of Pathology, Faculty of Health Sciences, University of the Witwatersrand, Johannesburg, South Africa; 7 Southern African Development Community Malaria Elimination Eight Secretariat, Windhoek, Namibia

**Keywords:** Dual active ingredient LLINs, Community effect, Malaria prevalence, Tanzania

## Abstract

**Background:**

Long-lasting insecticidal nets (LLINs) were once fully effective for the prevention of malaria; however, mosquitoes have developed resistance to pyrethroids, the main class of insecticides used on nets. Dual active ingredient LLINs (dual-AI LLINs) have been rolled out as an alternative to pyrethroid (PY)-only LLINs to counteract this. Understanding the minimum community usage at which these LLINs elicit an effect that also benefits non-users against malaria infection is important.

**Methods:**

We conducted a secondary analysis of a 3-year randomized controlled trial (RCT) in 84 clusters in North-western Tanzania to evaluate the effectiveness of three dual-AI LLINs: pyriproxyfen and alpha(α)-cypermethrin, chlorfenapyr and α-cypermethrin, and the piperonyl-butoxide (PBO) and permethrin compared to α-cypermethrin only LLINs. We measured malaria infection prevalence using 5 cross-sectional surveys between 2020 and 2022. We assessed net usage at the cluster level and malaria infection in children aged from 6 months to 14 years in 45 households per cluster. The trial was registered as a clinical trial on www.clinicaltrials.gov: ClinicalTrials.gov (NCT03554616) on 2018-06-13.

**Results:**

A total of 22,479 children from 12,654 households were tested for malaria using rapid diagnostic tests in January 2020, 2021, & 2022 and July 2020 & 2021. Among non-users, community-level usage of > 40% of dual-AI LLIN was significantly associated with protection against malaria infection: chlorfenapyr arm (OR: 0.44 (95% CI: 0.27–0.71), *p* = 0.0009), PBO arm (OR: 0.55 (95% CI: 0.33–0.94), *p* = 0.0277) and pyriproxyfen arm (OR: 0.61 (95% CI: 0.37–0.99), *p* = 0.0470) compared with non-users in clusters with > 40% usage of pyrethroid-only LLINs. There were indications of some protection against malaria infection to non-users in the chlorfenapyr arm when community-level usage was ≤ 40% (OR: 0.65 (95% CI: 0.42–1.01), *p* = 0.0528) compared to those living in clusters with > 40% usage of pyrethroid-only LLINs.

**Conclusion:**

Our study demonstrated that at a community usage of 40% or more of dual-AI LLINs non-users benefited from the presence of these nets. Noticeably, even when usage was ≤ 40% in the chlorfenapyr arm, non-users were better protected than non-users in the higher coverage pyrethroid-only arm. The greater difference in malaria risk observed between users and non-users indicates that LLINs play a crucial role in providing personal protection against malaria infection for the people using the net.

**Supplementary Information:**

The online version contains supplementary material available at 10.1186/s12889-025-21586-x.

## Background

Long-lasting insecticidal nets (LLINs) have been the core intervention for malaria control for many years and have contributed to a decline of > 25% global cases and > 66% global deaths since 2000 [1–4]. LLINs work by providing a barrier preventing female mosquitoes from taking bloodmeals from people sleeping under them and by killing or reducing a mosquito’s lifespan via the insecticide on the netting. The latter can result in a ‘community effect’ via which even non-users of nets benefit due to the reduced population of infectious mosquitoes [5–9]. This has been demonstrated in experimental hut trials [10–12], modelling [7, 13], and community trials [6, 14] where a higher level of community coverage of pyrethroid-only nets was associated with a decrease in malaria risk in those not using nets. Previous findings with pyrethroid-only nets have suggested that community coverage needs to be at least 15% and up to 85% before the community effect is realised [15]. However, this is a wide range to rely on for program implementation.

The community effect will depend on the insecticidal properties of the LLIN [16], as well as LLIN characteristics (coverage, netting integrity), and vector species behaviour (anthropophilic and zoophilic nature) [17]. Moreover, the presence of non-human alternative hosts, time spent indoors, under a net, and outdoors during peak biting hours, and mosquitoes insecticide resistance are also determinants of mass effect.

Insecticide resistance continues to be a threat to the effectiveness of pyrethroid-only LLINs [10, 16, 18–23]. In high insecticide resistance settings, the main mechanism of protection to people using pyrethroid-only nets is likely to be via the physical barrier of the net preventing mosquito bites- meaning non-users do not benefit to the same extent. Switching to novel malaria control tools, such as dual-active ingredient long-lasting insecticidal nets (dual-AI LLINs) will likely restore community effects in areas where the majority of vectors are resistant to pyrethroids [22].

Dual-AI LLINs have been shown to be more effective than pyrethroid-only nets, where mosquitoes are resistant to pyrethroids [24–29]. This is due to their unique modes of action: inhibiting the activity of the enzymes that breakdown pyrethroids in resistant vectors (piperonyl butoxide), sterilizing vectors that survive exposure to pyrethroids (pyriproxyfen), to disrupting the vectors’ ability to produce energy (chlorfenapyr), thereby restoring the effectiveness of LLINs against resistant mosquitoes [24–29]. Therefore, understanding the coverage levels required for community-wide effects is vital to help determine net coverage targets and plans for future campaigns.

Tanzania has one of the highest burdens of malaria cases and deaths [4]. In Tanzania, malaria is highest in the Lake Victoria zone. The core malaria intervention in the country is LLINs, which have been distributed widely in the country since 2007 [30]. Although Tanzania has made great efforts to implement LLINs in the general population, gaps in use, access, coverage, and ownership remain. As such, several distribution channels including mass campaigns, annual school net program (SNP), antenatal care (ANC) and the Expanded Programme on Immunization, and Targeted Replacement Campaign (TRC) are being implemented across the country. Dual-AI LLINs, particularly piperonyl-butoxide (PBO) LLINs, have been distributed in Tanzania through the SNP and ANC since 2018 in areas with high malaria burden, however, achieving high coverage of the population at risk remains a challenge. An understanding of the required level of coverage to achieve community benefits is key to the proper allocation of limited malaria control resources.

In this study, we assess malaria risk among users and non-users of nets living in areas with different community coverage of dual-AI LLINs as part of a secondary analysis of a large RCT assessing the impact of dual-AI LLIN in Misungwi, Tanzania [26].

## Methods

### Study site, design, and participants

Data used for this secondary analysis were collected in a 3-year, four parallel-arm cluster randomized controlled trial (cRCT) conducted on the southern border of Lake Victoria, Misungwi district (latitude 2°51’00.0"S, longitude 33°04’60.0” E), Mwanza region, in North-western Tanzania. The cRCT evaluated the effectiveness of three types of dual-active-ingredient long-lasting insecticidal nets compared to pyrethroid-only LLINs for reducing malaria. A total of 84 clusters were allocated to one of the four study arms (21 clusters per arm) using restricted randomisation (arms balanced on population size, baseline malaria prevalence, socioeconomic status, LLIN usage, and species composition). The four arms of the trial consisted of: Interceptor^®^ with only pyrethroid (PY) insecticide (alpha-cypermethrin, [control] arm), Interceptor^®^ G2 (chlorfenapyr LLIN (alpha-cypermethrin + chlorfenapyr), Royal Guard^®^ (pyriproxyfen LLIN (alpha-cypermethrin + pyriproxyfen), and Olyset™ Plus (permethrin + piperonyl-butoxide (PBO)). The main results from the cRCT has been previously published and showed that: chlorfenapyr LLINs showed superior efficacy over three years and piperonyl-butoxide LLINs over one year while pyriproxyfen LLINs did not seem to provide significant additional protection compared to pyrethroid-only LLIN [25, 26]. 

### Procedures

A total of 147,230 study LLINs were distributed (1 net for 2 persons) in 42,394 households as part of the trial between January 26 and January 28, 2019. In addition, there was continuous distribution of pyrethroid-only LLINs and PBO LLINs in the study area through ANC, and in September 2021 (33 months post-trial net distribution), 40,000 PBO LLINs were distributed by the local government across all study arms via SNP.

Malaria infection prevalence was measured during cross-sectional surveys at 12 months (January/February 2020), 18 months (July/August 2020), 24 months (January/February 2021), 30 months (July/August 2021), and 36 months (January/February 2022) post-intervention. At each survey timepoint, a random sample of 45 households in each cluster were selected. Up to two children aged between 6 months and 14 years in consenting households were randomly selected and tested for malaria infection using rapid diagnostic tests (RDT) (CareStart malaria HRP2 [pf], DiaSys, Wokingham, UK). A written informed consent was obtained from an adult guardian in each household interviewed and for selected children. In all consenting households, information (age, and sex) for all residents was recorded and all nets were visually examined by a trained interviewer, and the information about who (age and sex) used the net last night was recorded. Study net usage was then calculated as the percentage of all people (adults and children) who reported using study nets (i.e. chlorfenapyr, pyriproxyfen, and PBO LLINs) the previous night. Community/cluster study LLIN usage was calculated as the percentage of people (adults and children) within a given cluster at each survey point who reported using a dual-AI LLIN the previous night divided by the total number of observations in that cluster.

Data collection took place on smartphones using the Open-Data-Kit (ODK) software. Data from each field team was directly uploaded to a secure online database at the London School of Hygiene and Tropical Medicine (LSHTM) and the copy retained in Tanzania. After completion of the surveys, datasets were transferred to STATA release 15 (StataCorp, College Station, TX, USA) for further aggregation, cleaning, and analysis.

### Statistical analysis

The main outcome of interest was prevalence of malaria in non-users of nets comparing each of the dual-active-ingredient LLIN arms to the pyrethroid-only LLIN arm at 12 months, 18 months, 24 months, 30 months and 36 months post net distribution. The secondary outcomes were 1/determine at which level of community net usage can benefit non-users (elicit community effect), 2/ malaria risk differences between users and non-users at each survey timepoint.

Household socio-economic status (SES) indices were constructed based on self-reported ownership of certain goods (animals, poultry, phone, radio, bicycle, motorbike) and household possessions (including electricity, source of drinking water, toilet, number of sleeping rooms, type of cooking fuel). Principal component analysis (PCA) was used to develop a score which was then subdivided into wealth tertiles (lowest, middle, and highest) at the household level. Initially, malaria prevalence of users and non-users of nets was compared between study arms. House characteristics and structures (including roof, floor, eaves, walls, ceiling, and plastering, ) were not included in the construction of SES, instead, they were used to create a household design index and subdivided into tertiles (low-quality, medium-quality, and high quality).

To assess the community effect of the dual-AI LLINs (chlorfenapyr, pyriproxyfen, and PBO LLINs) relative to pyrethroid-only LLIN, the following analyses were conducted: 1/ comparison of malaria prevalence between users and non-users, and 2/ comparison of malaria prevalence in non-users in each dual-A.I. LLIN arm and non-users in the pyrethroid-only arm. Analyses 1 and 2 were done regardless of the cluster-level net usage. 3/ To assess whether the level of dual-AI LLIN cluster level usage had an impact on the non-users of net, a cut-off of 40% was selected based on findings of other studies, which indicated that usage levels of pyrethroid-only nets of between 30% and 50% were associated with community protection [7, 31], and to ensure a sufficient number of clusters in each category over time. We compared malaria prevalence in non-users living in clusters with > 40% or ≤ 40% community usage of dual-AI LLIN arms *versus* non-users living in pyrethroid-only clusters with community usage > 40% (Table 1). All malaria prevalence analyses used mixed-effect logistic regression. Model 1 and 2, included cluster as a random effect and fixed effects for survey timepoint, study arm, and adjusted for age group, sex, housing quality and socio-economic status (SES) and the baseline cluster-level variables used in restricted randomisation. Model 3, which combined all data, cluster and survey timepoint was included as random effect, while study arm categorised as ≤ 40% and > 40% cluster level usage as fixed effect and adjusting for the same variables included in model 1&2. The intervention effect was expressed as adjusted odds ratios (aORs). Differences in characteristics between user and non-users were compared using Chi-square (χ2 ), accounting for the clustered design. An interaction test between cluster-level usage (> 40% and ≤ 40% ) and survey timepoint was performed to test for the presence of effect modification.

**Table 1:** Comparison groups

Comparisons	Group1	Group 2
Analysis 1	users	non-users
Analysis 2	Non-users in dual-AI arms	Non-users in pyrethroid-only arm
Analysis 3	Non-users living in clusters with ≤ 40% community usage of study nets in dual-AI arms	Non-users living in clusters with > 40% community usage of study nets in pyrethroid-only arm

### Ethical considerations

Ethical approval for the RCT was obtained from the institutional review boards of the Tanzanian National Institute for Medical Research (reference number: NIMR/HQ/R.8a/Vol.IX/2743), Kilimanjaro Christian Medical University College (2267), London School of Hygiene and Tropical Medicine (14952), and University of Ottawa (H-05–19-4411).

### Role of the funding source

The funder of the study had no role in study design, data collection, data analysis, data interpretation, or writing of the report.

## Results

Between 07 January 2020 and 10 February 2022, five cross-sectional surveys were conducted in 12,654 randomly selected consenting households post-net distribution. Overall, 23% (*N* = 22479) [(14% (*N* = 4380) at 12 months, 22% (*N* = 4785) at 18 months, 20% (*N* = 4988) at 24 months, 30% (*N* = 3997) at 30 months, and 28% (*N* = 4329) at 36 months)] of children tested for malaria did not sleep under a net the previous night. Residents (all age group) not sleeping under study LLINs the previous night were more than doubled at 36 months (*n* = 14,940) compared to 5,975 at 12 months. Net use was highest in children under 5 years old (Table 1). In houses with not enough nets for every member (i.e. less than 1 net for every 2 people), boys over the age of 5 years were least likely to sleep under a net. People classified as highest SES were least likely to use nets. Net usage between girls and boys when under 5 years of age were similar (Table 2).

**Table 2:** Characteristics of non-users and users of the nets

Covariates	*n*	*n*
**Population (all age groups) in each study arms**		
Pyrethroid-only group	18,498	5361
Piperonyl butoxide group	16,854	6012
Pyriproxyfen group	17,462	6128
Chlorfenapyr group	19,081	6715
	**% users [95% CI]**,** n**	**% non-users[95% CI]**,** n**
**Child age and sex**		
0–4 years: Girls	85.8% [84.22,87.26], 3489	14.2% [12.74,15.78], 577
0–4 years: Boys	84.8% [83.38,86.16], 3538	15.2% [13.84,16.62], 633
5–9 years: Girls	77.5% [75.26,79.62], 3141	22.5% [20.38,24.74], 911
5–9 years: Boys	73.9% [71.81,75.84], 2983	26.1% [24.16,28.19], 1055
10–14 years: Girls	72.8% [70.40,75.11], 2218	27.2% [24.89,29.60], 828
10–14 years: Boys	66.0% [63.34,68.45], 2048	34.1% [31.55,36.66], 1058
**Household structure quality**		
Low quality	76.1% [74.46,77.72], 5814	23.9% [22.28,25.54], 1823
Medium quality	76.5% [74.31,78.49], 5783	23.5% [21.51,25.69], 1780
High quality	80.0% [78.07,81.72], 5820	20.0% [18.28,21.93], 1459
**Socio-economic status (SES)**		
Lowest	81.0% [79.22,82.71], 5995	19.0% [17.29,20.78], 1404
Middle	78.3% [76.51,80.07], 5730	21.7% [19.93,23.49], 1584
Highest	73.3% [71.25,75.25], 5692	26.7% [24.75,28.75], 2074
**Household with not enough coverage of study nets (1 net for 2 people) by child age group and sex**		
0–4 years: Girls	89.1% [87.33,90.71], 1764	10.9% [9.288,12.67], 215
0–4 years: Boys	88.4% [86.84,89.71], 1790	11.7% [10.29,13.16], 236
5–9 years: Girls	81.5% [79.25,83.53], 1721	18.5% [16.47,20.75], 391
5–9 years: Boys	77.1% [74.76,79.24], 1651	22.9% [20.76,25.24], 491
10–14 years: Girls	78.2% [75.79,80.48], 1286	21.8% [19.52,24.21], 358
10–14 years: Boys	70.4% [67.47,73.08], 1253	29.7% [26.92,32.53], 528

Following net distribution, overall mean malaria prevalence was 52% (*N* = 5062) in non-users [34% (*N* = 619) at 12 months, 58% (*N* = 1049) at 18 months, 52% (*N* = 998) at 24 months, 63% (*N* = 1202) at 30 months, and 46% (*N* = 1194) at 36 months]; and the mean malaria prevalence in users was 32% (*N* = 11845) [20% (*N* = 3761) at 12 months, 43% (*N* = 3736) at 18 months, 34% (*N* = 3990) at 24 months, 39% (*N* = 2795) at 30 months, 24% (*N* = 3135) at 36 months] (Additional file 1). A detailed summary of malaria prevalence among users of study nets, users of other nets, and non-users by study arm and survey timepoint is presented in Additional file 1.

In non-users, at 12 months, there was no evidence for lower malaria prevalence in any of the dual-AI LLIN arms compared to the pyrethroid-only arm. At 18 months, 24 months, and 30 months no difference in malaria prevalence was observed in the piperonyl-butoxide arm or pyriproxyfen arm compared with the pyrethroid-only arm (Table 3). In non-users living in the chlorfenapyr arm, there appeared to consistently be a reduction in prevalence compared to non-users in the pyrethroid-only arm. The odds of malaria infection were at least 40% lower for non-users living in the chlorfenapyr arm at 18 months [aOR 0.56 (95% CI 0.35–0.90), *p* = 0.0166], 24 months [aOR 0.55 (95% CI 0.29–1.03), *p* = 0.0621] and 30 months [aOR 0.57 (95% CI 0.33–0.96), *p* = 0.0353] compared to living in pyrethroid-only arm(Table 3). At 36 months, non-users in every dual-AI LLIN arm had lower malaria prevalence than non-users living in the pyrethroid-only arm (Table 3).

**Table 3:** Malaria prevalence in children (aged 6 months to 14 years) who are users and non-users at 12, 18, 24, 30 and 36 months surveys

Non-users analysis: *n* = 5,062				Users analysis: *n* = 17,417			
	*n*/*N* (%Prevalence)	aOR (95% CI)*	*p*-value	*n*/*N* (%Prevalence)	aOR (95% CI)*	*p*-value	*p*-value^‡^ for comparison between non-users and users
Pyrethroid-only group							
12 months	64/156 (41.0%)	1 (ref)		286/964 (29.7%)	1 (ref)		0.1444
18 months	154/236 (65.3%)	1 (ref)		488/992 (49.2%)	1 (ref)		0.0165
24 months	127/206 (61.7%)	1 (ref)		422/993 (42.5%)	1 (ref)		0.0208
30 months	174/258 (67.4%)	1 (ref)		333/698 (47.7%)	1 (ref)		0.0005
36 months	164/265 (61.9%)	1 (ref)		243/823 (29.5%)	1 (ref)		< 0.0001
Pyriproxyfen group							
12 months	63/178 (35.4%)	0.82 (0.41–1.60)	0.5584	169/891 (19.0%)	0.55 (0.36–0.83)	0.0046	0.0003
18 months	159/257 (61.9%)	0.91 (0.56–1.49)	0.7133	424/895 (47.4%)	0.87 (0.62–1.21)	0.3929	0.0012
24 months	128/261 (49.0%)	0.81 (0.43–1.51)	0.5004	344/997 (34.5%)	0.85 (0.59–1.24)	0.4045	0.0035
30 months	177/300 (59.0%)	0.71 (0.42–1.21)	0.2109	249/704 (35.4%)	0.63 (0.44–0.91)	0.0144	< 0.0001
36 months	131/293 (44.7%)	0.59 (0.37–0.95)	0.0284	171/757 (22.6%)	0.75 (0.49–1.13)	0.1628	< 0.0001
Piperonyl butoxide group							
12 months	38/127 (29.9%)	0.93 (0.46–1.89)	0.8431	168/941 (17.9%)	0.68 (0.45–1.01)	0.0580	0.0769
18 months	155/269 (57.6%)	0.85 (0.53–1.38)	0.5188	347/891 (39.0%)	0.67 (0.48–0.94)	0.0190	0.0001
24 months	146/260 (56.2%)	0.99 (0.54–1.84)	0.9811	366/999 (36.6%)	0.92 (0.64–1.33)	0.6625	< 0.0001
30 months	213/305 (69.8%)	1.40 (0.82–2.38)	0.2117	275/687 (40.0%)	0.80 (0.56–1.16)	0.2410	< 0.0001
36 months	154/320 (48.1%)	0.62 (0.40–0.97)	0.0361	182/727 (25.0%)	0.93 (0.62–1.40)	0.7413	< 0.0001
Chlorfenapyr group							
12 months	45/158 (28.5%)	0.66 (0.33–1.34)	0.2521	131/965 (13.6%)	0.39 (0.25–0.59)	< 0.0001	0.0005
18 months	144/287 (50.2%)	0.56 (0.35–0.90)	0.0166	364/958 (38.0%)	0.57 (0.41–0.80)	0.0009	0.0142
24 months	117/271 (43.2%)	0.55 (0.29–1.03)	0.0621	209/1001 (20.9%)	0.39 (0.27–0.58)	< 0.0001	< 0.0001
30 months	191/339 (56.3%)	0.57 (0.33–0.96)	0.0353	245/706 (34.7%)	0.64 (0.44–0.93)	0.0187	0.0001
36 months	105/316 (33.2%)	0.33 (0.20–0.53)	< 0.0001	156/828 (18.8%)	0.52 (0.35–0.79)	0.0020	0.0018

^a^aOR = adjusted odds ratio. Each intervention group is compared against the pyrethroid-only group for the same timepoint. Comparison p-value‡=p-value for comparison between users and non-net users by net type at each time point after adjusting for age, sex, socio-economic status (SES), and baseline cluster-level variables used in the restricted. Interaction between net type and survey: *p* = 0.3001 in non-net users

Across all study arms and survey timepoints, malaria infection was generally lower in users compared to non-users (Table 3). Malaria prevalence in non-users in the chlorfenapyr arm at each survey timepoint was similar or slightly higher than amongst users in the pyrethroid-only arm, i.e. the personal protection provided by users of pyrethroid-only nets was relatively similar to the protection provided by the community effect in the chlorfenapyr arm as shown in Additional file 3.

Table 4 presents the results of the impact of community usage on community effect by assessing malaria prevalence in non-users in clusters with above and below 40% study net usage. Cluster usage of dual-AI LLIN higher than 40% was associated with reduced odds of malaria infection in non-users living in the pyriproxyfen arm (aOR: 0.61 [95% CI: 0.37–0.99], *p* = 0.0470), PBO arm (aOR 0.55 [95% CI: 0.33–0.94], *p* = 0.0277); and chlorfenapyr arm (aOR 0.44 [95% CI: 0.27–0.71], *p* = 0.0009) compared with their counterparts living in clusters over 40% usage in the pyrethroid-only arm. There was also weak evidence of reduced odds of malaria infection in non-users living in the chlorfenapyr arm 55.1% (157/285) when community-level usage was ≤ 40% compared to those living in pyrethroid-only arm 45.7% (495/1083) when community usage was > 40%; aOR 0.65 [95% CI: 0.42–1.01]], *p* = 0.0528).

**Table 4:** Mean malaria prevalence in children (aged 6 months to 14 years) not using nets over three years in ≤ 40 and > 40% cluster level net usage

Covariates	Number of clusters^a^	%Prevalence in non-users (*n*/*N*)	aOR (95% CI), *p*-value*
> 40 coverage-pyrethroid-only group	69	55.1 (157/285)	1 (Ref)
≤ 40 coverage- pyrethroid-only group	36	62.9 (526/836)	1.24 (0.90–1.72), 0.1910
> 40 coverage- chlorfenapyr group	59	37.2 (107/288)	0.44 (0.27–0.71), 0.0009
≤ 40 coverage- chlorfenapyr group	46	45.7 (495/1083)	0.65 (0.42–1.01), 0.0528
> 40 coverage- piperonyl butoxide group	33	41.2 (77/187)	0.55 (0.33–0.94), 0.0277
≤ 40 coverage- piperonyl butoxide group	72	57.5 (629/1094)	1.26 (0.82–1.92), 0.2904
> 40 coverage- pyriproxyfen group	44	40.5 (106/262)	0.61 (0.37–0.99), 0.0470
≤ 40 coverage- pyriproxyfen group	61	53.8 (552/1027)	1.01 (0.66–1.54), 0.9726

^a^Number of clusters: total number of clusters contributing to the category over the study period

*aOR adjusted odds ratio. Adjusted for age, sex, SES, survey timepoint, and baseline cluster-level variables (net usage, malaria prevalence)

A sensitivity analysis was conducted using the same dataset, excluding 36 months survey (as new PBO LLINs were distributed in all the clusters through SNP a few months before the survey). In this analysis, non-users in chlorfenapyr arm (aOR 0.48; 95% CI: 0.29–0.79, *p* = 0.0040) when cluster level usage was > 40% were significantly protected against malaria compared to non-users in > 40% cluster usage in pyrethroid-only arm; and very weak evidence with piperonyl-butoxide arm (aOR 0.63; 95% CI: 0.37–1.07, *p* = 0.0895) and pyriproxyfen arm (aOR 0.63; 95% CI: 0.38–1.04, *p* = 0.0711). No statistically significant protection was observed in all the three dual-AI LLINs when coverage was below or equal to 40% after excluding 36 months in the analysis.

Overall 83 out of the 84 (99%) clusters had > 40% usage at 12 months, this reduced to 75% (*n* = 63) at 18 months, 50% (*n* = 42) at 24 months, 18% (*n* = 15) at 30 months and only 2% (*n* = 2) at 36 months (Additional file 2).

To see if the effect of community usage was modified by the survey period, we examined for an interaction between cluster level usage and survey timepoint. The test for interaction between levels of community dual-AI LLIN usage and survey time point showed no difference in the effect of community dual-AI LLIN usage on the odds of malaria infection among children who did not use nets (*p* = 0.3092).

## Discussion

This is a secondary analysis of a cluster randomised trial of dual-AI LLINs assessing the community effect of three dual-AI LLINs (chlorfenapyr LLINs, pyriproxyfen LLINs, and PBO LLINs) compared with pyrethroid -only LLIN. Users were always more protected than non-users regardless of net type and survey timepoint, underscoring the importance of personal protection provided by nets, even in areas of resistant mosquitoes. In addition, regardless of community usage levels, non-users living in the chlorfenapyr arm were more protected than non-users in the pyrethroid-only arm. We also found that cluster usage of dual-AI LLIN above 40% provided significantly better protection against malaria infection to non-users compared to non-users living in the pyrethroid-only arm, suggesting there was less of a community effect in the pyrethroid-only arm. There was borderline evidence of chlorfenapyr LLINs still providing better community protection to non-users, even when community usage was ≤ 40% compared to pyrethroid-only LLINs when cluster usage is > 40%. After excluding 36 month data, non-users in all dual-AI LLINs when cluster level usage was > 40% benefited from their presence, but this was more pronounced in chlorfenapyr arm than the rest.

An early review by Lines et al. [15] identified 21 studies assessing the community effect of pyrethroid-only LLINs and reported a wide range of minimum community coverage levels (from 15 to 85%) which lead to community effects protecting people sleeping without nets. Consistent with this, a study conducted in Kenya [32] concluded that at least 35% community coverage of nets is required to protect people sleeping without nets, while another study [6] reported that residents not using nets and living within 300 m from a community using insecticide-treated nets (usage greater than 50%) were protected against malaria compared to those further away. Meanwhile, Lindblade et al. [33] found that community net usage (> 82%) protected both users and non-users equally. Models have also been used to estimate the threshold of community net usage necessary to elicit a community effect. For example, Killeen et al. [7] modelled that coverage of 35–65% would be needed to achieve community-wide benefits. Another model [23] suggested that as soon as one person uses an LLIN, there is a small indirect impact on non-users (even if marginal) compared to a hypothetical scenario where nobody is using an LLIN. All models suggest that the benefits for both users and non-users increase with net usage.

The present study adds to the body of existing evidence and demonstrates that when a net is very effective, as observed for chlorfenapyr LLINs, both users and non-users are protected even at moderate to low levels of community coverage. With less effective nets such as piperonyl-butoxide LLIN and pyriproxyfen LLINs, the impact on non-users was not as evident. Up to 30 months, there was no difference in malaria prevalence between non-users in PBO arm and pyrethroid-only arm suggesting limited community protection from PBO LLINs except when PBO LLIN cluster level usage was above 40%. Greater and longer-lasting efficacy has been observed with this class of nets in two other cRCTs [27, 28]. Although neither of these trials specifically examined the impact of the net on non-users, in Uganda, the effect of PBO LLIN on malaria prevalence was more pronounced when only children using PBO LLIN (per protocol) were considered, rather than including all children regardless of net usage status (intention to treat) [28], suggesting a small or no community effect. However, the cluster level usage coverage of PBO LLIN is not reported so this is difficult to say with confidence. In another study in Tanzania (Muleba), during the first two years of follow-up, a similar reduction in malaria prevalence was observed for intention-to-treat and per protocol analyses, indicating that both non-users and users may have been protected equally by PBO LLIN [27]. In the third year, however, PBO LLIN showed reduced prevalence among users, as net usage and efficacy declined [34]. However in the Muleba trial, usage of PBO LLIN during the first two years of the study was higher (from 79% in the first year and 54% in the second year) than reported in the present study (74% in the first year and 30% in the second year) and could explain the difference of impact.

Pyriproxyfen LLINs were designed to provide a community effect through sterilizing vectors as well as reducing the lifespan of female vectors after they have blood-fed [35] and survived exposure to the insecticide on the net. Pyriproxyfen LLINs seem to have had some impact on malaria indicators in another trial conducted in Burkina Faso [29]. In the present trial, malaria prevalence was reduced in users only at 12 months compared to people using pyrethroid-only LLINs. Consistent to PBO LLIN results, no low coverage in the pyriproxyfen arm did not benefit non-users.

It is worth noting in the present study that 36 months after distribution, individuals not using nets in all the dual-AI arms had lower odds of infection compared to those not using nets in the pyrethroid-only arm. This impact was likely associated with the distribution of new PBO LLINs across all arms four months before the 36-month survey, which increased the usage of new nets and effective nets. In addition, PBO may enhance the efficacy of pyriproxyfen as it does for pyrethroid as these two insecticides may have similar mechanisms of resistance [36]. However, even after we excluded 36 months data into the analysis, non-users in all dual-AI arms had reduced malaria infection and the effect was more evident in chlorfenapyr arm. This provided evidence that increase in cluster level usage in the dual-AI arms above 40% will likely elicit stronger community protection.

Regardless of the impact of the nets on non-users, using any net was always more protective against malaria prevalence than not using one. This was observed for all net types including the pyrethroid-only LLIN. This result is consistent with other studies that reported higher malaria prevalence or incidence in non-users compared to users sleeping under standard pyrethroid-only LLINs even in areas with pyrethroid resistance and highlights the importance of the barrier effect of the nets [37–41].

Furthermore, high usage (> 40%) clusters were unsurprisingly concentrated in the timepoints closest to the distribution of nets implying that the majority of the nets in this category were new nets (with fresh insecticide), whilst, in the later years, the majority of the clusters were concentrated in ≤ 40% category and likely to be older nets.

This study has several limitations. The study was not designed for this secondary analysis and may have insufficient power to adequately assess separately the impact on users and non-users using multiple testing that would lead other results occurring by chance. Net usage was estimated on information provided by households’ members which might not always be reliable. Finally, non-users were defined as people (children and adults) who did not sleep under any net the night before and might not capture occasional net usage during the week which also may influence the conclusions.

Regardless, this secondary analysis provides insight into the efficacy of these novel dual-AI LLINs within a region characterized by moderate to high malaria transmission and high resistance to pyrethroids. In settings with limited resources and the presence of insecticide resistance, the deployment of an effective net, such as chlorfenapyr LLINs, even at suboptimal coverage, could be considered as it would be more effective and even more cost-effective [26] than high coverage of pyrethroid-only LLINs. This aligns with previous modelling work [42] which emphasized that a massive reduction in mosquitoes would be more important than coverage alone. However, even the most effective net in this study did not produce a sufficient reduction in mosquitoes to prevent users of these nets from being exposed to high levels of malaria infection. A key message was that users were always better protected than non-users and therefore after providing the most effective nets, national malaria control program could consider maximizing usage for better impact. Finally, as observed by other studies [43–48] pyrethroid-only nets still provided some protection in this area of pyrethroid resistance. Non-users of nets in clusters where chlorfenapyr nets were used were similarly protected as users in clusters where pyrethroid only nets were used. As malaria was still high even amongst users of dual-AI LLINs, meaning that these nets did not adequately control malaria and infection prevalence. New, more effective vector control tools like the indoor residual spraying (IRS), attracted targeted sugar baits (ATSBs), spatial repellents (SRs) [49] are therefore urgently needed to complement nets in providing better protection against malaria than the protection provided by nets alone.

## Conclusion

In areas where resistance to pyrethroids is prevalent in malaria vectors, chlorfenapyr LLINs offer enhanced protection to individuals who use them as well as those who do not, even at lower coverage levels. This added protection for non-users is could also be attained with nets containing piperonyl-butoxide (PBO) and pyriproxyfen when the overall cluster usage exceeds 40%. Users were more protected than non-users and emphasized the necessity to optimize net usage to benefit from their full potential. Nonetheless, in regions facing constrained financial resources and insecticide resistance, the distribution of the most effective net could be considered as an alternative to high-population coverage of conventional nets. This strategic allocation would ensure maximal impact in the control of malaria despite limitations in resources and resistance challenges.

## Supplementary Information

Additional file 1.

Additional file 2.

## Data Availability

The datasets generated and/or analysed during the current study are not publicly available due strict laws in Tanzania that restrict data to be shared outside the country without a Data Transfer agreement (DTA.pdf (nimr.or.tz)). A DTA process will need to be completed by interested researchers in contact with Mr Eliud Lukole (Eliud.Lukole@lshtm.ac.uk) and Prof. Natacha Protopopoff (Natacha.Protopopoff@lshtm.ac.uk).
