# Supplementary material for: Community benefits of mass distribution of three types of dual-active-ingredient long-lasting insecticidal nets against malaria prevalence in Tanzania: evidence from a 3-year cluster-randomized controlled trial
Source: BMC Public Health. 2025 Jan 28;25:346. doi: 10.1186/s12889-025-21586-x (PMC11773712; doi:10.1186/s12889-025-21586-x)
Supplement: Supplementary file 1 — Additional file 1. [file 12889_2025_21586_MOESM1_ESM.docx]

Additional file 1: Malaria prevalence in non-net users and users (of study nets and other nets)

| **Study arm** | **Covariates** | **12 months** | **18 months** | **24 months** | **30 months** | **36 months** |
| --- | --- | --- | --- | --- | --- | --- |
| pyrethroid-only group | Users of other nets | 29.3% (78/266) | 47.6% (155/326) | 40.9% (164/401) | 44.5% (138/310) | 27.3% (133/488) |
|  | Users of study nets | 29.8% (208/698) | 50.0% (333/666) | 43.7% (258/591) | 50.3% (195/388) | 32.8% (110/335) |
|  | Non-net users | 41.0% (64/156) | 65.3% (154/236) | 61.7% (127/206) | 67.4% (174/258) | 61.9% (164/265) |
| chlorfenapyr group | Users of other nets | 9.2% (21/228) | 37.0% (104/281) | 19.5% (77/394) | 35.3% (122/346) | 18.8% (96/510) |
|  | Users of study nets | 14.9% (110/737) | 38.4% (260/677) | 21.8% (132/607) | 34.2% (123/360) | 18.9% (60/318) |
|  | Non-net users | 28.5% (45/158) | 50.2% (144/287) | 43.2% (117/271) | 56.3% (191/339) | 33.2% (105/316) |
| piperonyl butoxide group | Users of other nets | 13.7% (40/293) | 37.2% (148/398) | 34.9% (214/614) | 36.1% (181/501) | 25.4% (149/587) |
|  | Users of study nets | 19.8% (128/648) | 40.4% (199/493) | 39.4% (152/386) | 50.5% (94/186) | 23.6% (33/140) |
|  | Non-net users | 29.9% (38/127) | 57.6% (155/269) | 56.2% (146/260) | 69.8% (213/305) | 48.1% (154/320) |
| pyriproxyfen group | Users of other nets | 17.8% (44/247) | 50.3% (174/346) | 33.8% (179/529) | 34.9% (139/398) | 20.9% (118/565) |
|  | Users of study nets | 19.4% (125/644) | 45.5% (250/549) | 35.3% (165/468) | 36.0% (110/306) | 27.6% (53/192) |
|  | Non-net users | 35.4% (63/178) | 61.9% (159/257) | 49.0% (128/261) | 59.0% (177/300) | 44.7% (131/293) |
| Overall | Users of other nets | 17.7% (183/1034) | 43.0% (581/1351) | 32.7% (634/1938) | 37.3% (580/1555) | 23.1% (496/2150) |
|  | Users of study nets | 20.9% (571/2727) | 43.7% (1042/2385) | 34.5% (707/2052) | 42.1% (522/1240) | 26.0% (256/985) |
|  | Non-net users | 33.9% (210/619) | 58.3% (612/1049) | 51.9% (518/998) | 62.8% (755/1202) | 46.4% (554/1194) |

Additional file 2: Distribution of clusters in each community usage category (≤ 40% and > 40%) per time point per study arm

| Covariates | 12 months | 18 months | 24 months | 30 months | 36 months |
| --- | --- | --- | --- | --- | --- |
| >40 coverage-**p**yrethroid**-only group** | 21 | 19 | 20 | 7 | 2 |
| ≤40 coverage- **p**yrethroid**-only group** | 0 | 2 | 1 | 14 | 19 |
| >40 coverage- chlorfenapyr group | 21 | 16 | 15 | 7 | 0 |
| ≤40 coverage- chlorfenapyr group | 0 | 5 | 6 | 14 | 21 |
| >40 coverage- piperonyl butoxide group | 21 | 11 | 1 | 0 | 0 |
| ≤40 coverage- piperonyl butoxide group | 0 | 10 | 20 | 21 | 21 |
| >40 coverage- pyriproxyfen group | 20 | 17 | 6 | 1 | 0 |
| ≤40 coverage- pyriproxyfen group | 1 | 4 | 15 | 20 | 21 |
| **Total clusters** | 84 | 84 | 84 | 84 | 84 |

Additional file 3: Comparisons of malaria infection between users of pyrethroid-only LLIN and non-users in chlorfenapyr arm by survey timepoint

| **survey** | **Non-users in chlorfenapyr group: n/N (%Prevalence)** | **Users in pyrethroid-only group: n/N (%Prevalence) *** | **aOR** | **95% CI** | **p-value** |
| --- | --- | --- | --- | --- | --- |
| 12 months | 45/158 (28.5%) | 286/964 (29.7%) | 0.85 | 0.45-1.59 | 0.6022 |
| 18 months | 144/287 (50.7%) | 488/992 (49.2%) | 0.92 | 0.63-1.32 | 0.6405 |
| 24 months | 117/271 (43.2%) | 422/993 (42.5%) | 0.77 | 0.48-1.25 | 0.2902 |
| 30 months | 191/339 (56.3%) | 333/698 (47.7%) | 1.01 | 0.65-1.58 | 0.9676 |
| 36 months | 105/316 (33.2%) | 243/823 (29.5%) | 0.83 | 0.48-1.42 | 0.4872 |
| *reference arm/group/variable | | | | | |
