# Supplementary material for: Community benefits of mass distribution of three types of dual-active-ingredient long-lasting insecticidal nets against malaria prevalence in Tanzania: evidence from a 3-year cluster-randomized controlled trial
Source: BMC Public Health. 2025 Jan 28;25:346. doi: 10.1186/s12889-025-21586-x (PMC11773712; doi:10.1186/s12889-025-21586-x)
Supplement: Supplementary file 2 — Additional file 2. [file 12889_2025_21586_MOESM2_ESM.zip › MoshaJF_2023_dual ai LLINs_3years.pdf]

Effectiveness of long-lasting insecticidal nets with pyriproxyfen–pyrethroid, chlorfenapyr–pyrethroid, or piperonyl butoxide–pyrethroid versus pyrethroid only against malaria in Tanzania: final-year results of a four-arm, single-blind, cluster-randomised trial

Jacklin F Mosha, Nancy S Matowo*, Manisha A Kulkarni, Louisa A Messenger, Eliud Lukole, Elizabeth Mallya, Tatu Aziz, Robert Kaaya, Boniface A Shirima, Gladness Isaya, Monica Taljaard, Ramadhan Hashim, Jacklin Martin, Alphaxard Manjurano, Immo Kleinschmidt, Franklin W Mosha, Mark Rowland, Natacha Protopopoff*

Summary

Background New classes of long-lasting insecticidal nets (LLINs) containing two active ingredients have been recently recommended by WHO in areas where malaria vectors are resistant to pyrethroids. This policy was based on evidence generated by the first 2 years of our recently published trial in Tanzania. In this Article, we report the final third-year trial findings, which are necessary for assessing the long-term effectiveness of new classes of LLIN in the community and the replacement intervals required.

Methods A third year of follow-up of a four-arm, single-blind, cluster-randomised controlled trial of dual active ingredient LLINs was conducted between July 14, 2021, and Feb 10, 2022, in Misungwi, Tanzania. Restricted randomisation was used to assign 84 clusters to the four LLIN groups (1:1:1:1) to receive either standard pyrethroid (PY) LLINs (reference), chlorfenapyr–PY LLINs, pyriproxyfen–PY LLINs, or piperonyl butoxide (PBO)–PY LLINs. All households received one LLIN for every two people. Data collection was done in consenting households in the cluster core area with at least one child between 6 months and 15 years of age who permanently resided in the selected household. Exclusion criteria were householders absent during the visit, living in the cluster buffer area, no adult caregiver capable of giving informed consent, or eligible children who were severely ill. Field staff and study participants were masked to allocation, and those analysing data were not. The primary 24-month endpoint was reported previously; here, we present the secondary outcome, malaria infection prevalence in children at 36 months post LLIN distribution, reported in the intention-to-treat analysis. The trial was registered with ClinicalTrials.gov (NCT03554616) and is now complete.

Findings Overall usage of study nets was 1023 (22·3%) of 4587 people at 36 months post distribution. In the standard PY LLIN group, malaria infection was prevalent in 407 (37·4%) of 1088 participants, compared with 261 (22·8%) of 1145 in the chlorfenapyr–PY LLIN group (odds ratio 0·57, 95% CI 0·38–0·86; p=0·0069), 338 (32·2%) of 1048 in the PBO–PY LLIN group (0·95, 0·64–1·42; p=0·80), and 302 (28·8%) of 1050 in the pyriproxyfen–PY LLIN group (0·82, 0·55–1·23; p=0·34). None of the participants or caregivers reported side-effects.

Interpretation Despite low coverage, the protective efficacy against malaria offered by chlorfenapyr–PY LLINs was superior to that provided by standard PY LLINs over a 3-year LLIN lifespan. Appropriate LLIN replacement strategies to maintain adequate usage of nets will be necessary to maximise the full potential of these nets.

Funding Department for International Development, UK Medical Research Council, Wellcome Trust, Department of Health and Social Care, and Bill & Melinda Gates Foundation via the Innovative Vector Control Consortium.

Copyright © 2023 The Author(s). Published by Elsevier Ltd. This is an Open Access article under the CC BY 4.0 license.

Lancet Infect Dis 2023

Published **Online** September 27, 2023 https://doi.org/10.1016/S1473-3099(23)00420-6

See **Online/Comment** https://doi.org/10.1016/S1473-3099(23)00437-1

*Joint first authors

Department of Parasitology, National Institute for Medical Research, Mwanza Medical Research Centre, Mwanza, Tanzania (J F Mosha PhD, E Lukole MSc, T Aziz BSc, R Hashim MSc, J Martin MSc, A Manjurano PhD); **Department of Disease Control** (N S Matowo PhD, L A Messenger PhD, Prof M Rowland PhD, N Protopopoff PhD) **and Medical Research Council Tropical Epidemiology Group, Department of Infectious Disease Epidemiology** (Prof I Kleinschmidt PhD), **London School of Hygiene and Tropical Medicine, London, UK; School of Epidemiology and Public Health, University of Ottawa, Ottawa, ON, Canada** (M A Kulkarni PhD, M Taljaard PhD); **Department of Environmental and Occupational Health, School of Public Health, University of Nevada, Las Vegas, NV, USA** (L A Messenger); **Department of Parasitology, Kilimanjaro Christian Medical University College, Moshi, Tanzania** (E Mallya BSc, R Kaaya BSc, B A Shirima BSc, G Isaya BSc, J Martin, Prof F W Mosha PhD); **Ottawa Hospital Research Institute, Ottawa, ON, Canada** (M Taljaard); **Wits Research Institute for Malaria, School of Pathology, Faculty of Health**

Introduction

Pyrethroid (PY) long-lasting insecticidal nets (LLINs) are the primary method of malaria control in sub-Saharan Africa.[1] These nets have contributed considerably to the decline in malaria morbidity and all-cause mortality across the region. However, the rapid spread of PY resistance in malaria vector populations[2] is a contributing factor to the stagnation of decline in malaria cases and mortality in recent years.

To counter the challenge of PY resistance, several new classes of LLINs have been developed that include a mixture of a PY insecticide and the synergist piperonyl butoxide (PBO) or a second insecticide with a differing mode of action to PYs. The most recent

Sciences, University of the Witwatersrand, Johannesburg, South Africa (Prof I Kleinschmidt); Southern African Development Community Malaria Elimination Eight Secretariat, Windhoek, Namibia (Prof I Kleinschmidt)

Correspondence to: Nancy S Matowo, Department of Disease Control, London School of Hygiene and Tropical Medicine, London WC1E 7HT, UK nancy.matowo@lshtm.ac.uk

Research in context

Evidence before this study

On Jan 20, 2023, we searched PubMed using the terms "malaria", "malaria control", "efficacy/effectiveness/effect", "randomized controlled trial", "net" "insecticide-treated net" or "long-lasting insecticidal net" or "net" combined with "chlorfenapyr", "pyriproxyfen", and "piperonyl butoxide", for published abstracts and articles in English since March 26, 2022, when we published our main randomised controlled trial 2-year findings. We identified two cluster-randomised trials reporting the effectiveness of long-lasting insecticidal nets (LLINs) with piperonyl butoxide–pyrethroid (PBO–PY), pyriproxyfen–PY, and chlorfenapyr–PY on epidemiological and entomological outcomes. The first trial, carried out in Uganda, reported the final-year results of the effect of PBO–PY LLINs compared with standard PY LLINs on malaria outcomes. In the Uganda trial, at 25 months after implementation of LLINs, PBO–PY LLINs were shown to provide additional protection against malaria infection prevalence in children aged 2–10 years compared with standard LLINs in areas of high-level PY resistance. The malaria infection prevalence was 20% lower in the PBO–PY LLIN group compared with the standard LLIN group after 25 months of net use in the field. The systematic review on the effectiveness of PBO–PY LLNs concluded that in areas with moderate to high levels of PY resistance, PBO–PY LLINs are superior to standard PY LLINs in malaria outcomes at 21–25 months of community use. The second trial was conducted in Benin, assessing the efficacy of two new classes of pyriproxyfen–PY LLINs and chlorfenapyr–PY LLINs compared with standard PY LLINs on malaria case incidence and entomological outcomes at 24 months after deployment. In Benin, the authors reported a 46% reduction in malaria case incidence in children who received chlorfenapyr–PY LLINs compared with those in the standard PY group. There was a 58% reduction in entomological inoculation rates in the pyriproxyfen–PY LLIN group compared with standard PY LLINs, and no additional effect of the pyriproxyfen–PY LLINs was evident on epidemiological indices at 24 months post distribution.

Added value of this study

To the best of our knowledge, this trial reports, for the first time, the effectiveness of chlorfenapyr–PY LLINs and pyriproxyfen-PY LLINs during 3 years of community use, the lifespan recommended by WHO. Chlorfenapyr–PY LLINs were still more effective even at low usage than standard PY LLINs against malaria outcomes in an area with PY-resistant mosquitoes after 3 years of community use. However, in our trial, as already reported in the 2-year findings, PBO–PY LLINs lasted for only 12 months and there was no additional protection given by PBO–PY LLINs in the third year of trial follow-up. We did not observe any additional protection effect of pyriproxyfen-PY LLINs on malaria prevalence and entomological indices at 36 months after deployment.

Implications of all the available evidence

The trial findings are important for WHO when making policy recommendations on the performance and lifespan of novel classes of LLINs containing dual active ingredients with different modes of action from PYs. Following the 2-year trial findings in Tanzania and Benin, on March 14, 2023, WHO recommended chlorfenapyr-based LLINs and pyriproxyfen–PY LLINs to prevent malaria in adults and children in areas with pyrethroid-resistant mosquitoes. The third-year results demonstrated that with improved durability and community usage, chlorfenapyr–PY LLINs could sustain malaria control in areas with PY-resistant vectors over 3 years. To preserve the efficacy of chlorfenapyr-based LLINs, appropriate resistance management strategies must be in place.

developments encompass two new classes of nets: one with a combination of a PY and pyriproxyfen (an insect growth regulator that sterilises adult female mosquitoes), and the other with a mixture of a PY and the pyrrole chlorfenapyr (an insecticide that disrupts the ability of insects to produce and supply adequate energy to the flight muscles). The results of several randomised controlled trials evaluating these new classes of nets have indicated that some of these new LLINs were more effective than standard PY LLINs on malaria outcomes.[3–6]

The first 2 years of results of the present study, which were reported previously,[4] demonstrated that chlorfenapyr–PY LLINs provided better protection over 24 months compared with standard PY LLINs on malaria epidemiological and entomological outcomes. The superior protection provided by PBO–PY LLINs compared with standard PY LLINs lasted for 1 year, whereas there was weak evidence of a reduction in malaria prevalence and vector density for pyriproxyfen–PY LLINs during the first year.[4] At 24 months post distribution, malaria infection was prevalent in 549 (45·8%) of 1199 people in the reference group, compared with 472 (37·5%) of 1258 for pyriproxyfen–PY LLINs (odds ratio [OR] 0·79, 95% CI 0·54–1·17; p=0·24), 512 (40·7%) of 1259 for PBO–PY LLINs (0·99, 0·67–1·45, p=0·96), and 326 (25·6%) of 1272 for chlorfenapyr–PY LLINs (0·45, 0·30–0·67, p=0·0001).[4] The same brands of chlorfenapyr–PY LLIN and pyriproxyfen–PY LLIN have been evaluated in Benin[6] in an area with different malaria transmission dynamics, vector species composition, and insecticide resistance intensity, with similar findings to our study. On the basis of the 2-year results of these two randomised controlled trials,[4,6] WHO has issued a recommendation for the deployment of chlorfenapyr–PY LLINs for malaria control in areas with PY resistance, and has given pyriproxyfen–PY LLINs a conditional recommendation.[7–9]

However, additional evidence is necessary to support the appropriate frequency of roll-out of each type of net. According to WHO, LLINs are expected to demonstrate insecticide biological efficacy for 20 washes in the laboratory and 3 years of use in the community.[10] National malaria control strategies and funding cycles for net replacement usually follow a 3-year cycle, but several community studies have shown that this interval is not appropriate for maintaining maximum effective coverage of nets, as usage drops quickly due to accumulating wear and tear in LLIN textiles.[11–13] A study conducted in northwest Tanzania found that only 29% of PBO–PY LLINs and 26% of PY LLINs remained in the study area 3 years post distribution.[14] There was some indication, however, that despite an increase in malaria prevalence over the 3 years in the PBO–PY LLIN group, malaria prevalence was still lower for people using these nets compared with those sleeping under a standard PY LLIN.[15] Furthermore, although those new classes of nets have met the WHO bioefficacy criteria in the laboratory,[16] they still need to demonstrate long-lasting bioefficacy in the community, and there is some evidence that the second partner insecticide or synergist might not last for 3 years.[14,17] It is, therefore, important to assess whether the superior effectiveness of new classes of LLINs versus standard PY LLINs is sustained over 3 years, as it will have cost implications for the roll-out and replacement strategies of these nets.

Herein, we report the third-year results of a cluster-randomised trial evaluating the effectiveness of chlorfenapyr–PY LLINs, pyriproxyfen–PY LLINs, and PBO–PY LLINs compared with PY LLINs against community malaria infection and transmission in an area of intense PY resistance in northwest Tanzania.

Methods

Study design and setting

We conducted a parallel four-arm, single-blind, superiority cluster-randomised trial in 84 study clusters in Misungwi district, northwest Tanzania, from Aug 31, 2018, to Feb 15, 2022. The results of the 2-year primary endpoints have been published previously.[4] The study design and setting have been detailed elsewhere.[18] In brief, at baseline in 2018, the study area had a moderate malaria transmission, with prevalence of 44·2%, and an entomological inoculation rate of 4·4 infective bites per house per month, mainly transmitted by *Anopheles funestus*.[19] Each cluster had an inner core area and a minimum 600 m buffer between clusters to reduce the risk of contamination. Clusters were initially randomly allocated in a 1:1:1:1 ratio to Royal Guard (mixture of pyriproxyfen–PY LLIN; Disease Control Technologies, Greer, SC, USA), Interceptor G2 (mixture of chlorfenapyr–PY; BASF SE, Ludwigshafen, Germany), Olyset Plus (mixture of PBO–PY LLIN; Sumitomo Chemical, Tokyo, Japan), or the reference group Interceptor (PY LLIN; BASF SE). All populations living in study clusters were eligible to receive the study LLINs, but only those living in the core area of each cluster were included in the follow-up.

Ethical approval was obtained from the institutional review boards of the Tanzanian National Institute for Medical Research (NIMR/HQ/R.8a/Vol.IX/2743), Kilimanjaro Christian Medical University College (2267), London School of Hygiene & Tropical Medicine (14952), and the University of Ottawa (H-05-19-4411). Written informed consent, in Kiswahili, was obtained from village leaders and household heads for trial participation and data collection before each activity.

Participants

Repeated cross-sectional surveys were conducted at 12 months, 18 months, and 24 months (primary endpoint); these results have been published previously.[4] The 30-month survey was conducted between July 14 and Aug 16, 2021, during the high malaria transmission season, and the 36-month survey between Jan 12 and Feb 10, 2022, in the low transmission season. All households in the core area of each cluster with at least one child between 6 months and 14 years of age who permanently resided in the selected household, and an adult caregiver who could provide written consent, were eligible to be included in the surveys. Exclusion criteria were dwellings not found or vacant, no adult caregiver capable of giving informed consent, or eligible children who were severely ill. At each timepoint, 45 households were randomly selected (by simple random sampling without replacement using a random number generator) from each cluster, using the census list compiled during baseline enumeration. In each house, a maximum of two children of appropriate age were selected for detection of malaria parasitaemia. Mosquito monitoring was done every 3 months in eight households randomly selected from the core area of each cluster.

Randomisation and masking

Each of the 84 clusters was allocated to one of the four study groups by an independent statistician using the method of covariate constrained randomisation,[18] ensuring that clusters were balanced on the following covariates: population size, malaria infection prevalence in the baseline year, socioeconomic status, LLIN usage, and cluster suitability for *Anopheles gambiae* sensu stricto (ss), and for *A funestus*.[18] Approximately 200 000 random allocations were generated using Stata 11 and tested against the balancing criteria, and one acceptable allocation was selected at random.

All nets were similar in appearance, except that each of the four types of nets had a different colour-coded loop and a unique identifying number. Study participants and field staff were masked to the type of net that was allocated to a cluster. The analysis reported in the present paper was performed unmasked.

Procedures

A mass distribution of the four types of LLIN was conducted in January, 2019, delivered free of charge to all populations living in the study area. Study nets were monitored every year for chemical contents.[18] During the study period, standard PY LLINs were provided by the government to pregnant women and infants during antenatal and vaccination services in health facilities. Yearly school net distributions were suspended for the first 30 months of the study but resumed in October, 2021, when children in grades 1, 3, 5, and 7 attending primary schools received one PBO–PY LLIN each.

During the cross-sectional household surveys, information on household characteristics and LLIN ownership and use (study LLIN and others) was collected using electronic forms in tablets with Open Data Kit. For every selected child, malaria infection was tested using malaria rapid diagnostic tests (CareStart RDTs; HRP2 [pf], DiaSys, Wokingham, UK), haemoglobin was measured (HemoCue Hb 201+, Aktiebolaget Leo Diagnostics, Helsingborg, Sweden), temperature was taken, and history of fever during the preceding 48 h was recorded.

Vector population density was monitored on a quarterly basis between Jan 7 and Dec 6, 2021, across all study clusters using US Centers for Disease Control and Prevention (CDC) light traps. 13 houses were randomly selected from the core area of each cluster and up to eight houses were used for indoor mosquito collections. CDC light traps were installed in bedrooms at the foot of the bed, and occupants' nets were replaced by the standard PY LLIN during the night of collection. CDC light traps were set between 1800 h and 0630 h.

All sampled mosquitoes were separated by sex, and females were identified morphologically separating Culicine and Anopheline genera. *Anopheles* were further separated between the *A gambiae* and *A funestus* complexes, and other anophelines species. A subset of ten *Anopheles* per household per species were analysed for the presence of *Plasmodium falciparum* circumsporozoite protein using enzyme-linked immunosorbent assays. Real-time PCR TaqMan assays were performed to differentiate sibling species of *A gambiae* sensu lato (sl) and *A funestus* sl.

Outcomes

The main third-year endpoint was malaria infection prevalence in children aged 6 months to 14 years at 36 months post LLIN distribution. Other outcomes were malaria infection prevalence at 30 months, severe anaemia in children under 5 years old (haemoglobin <8 g/dL) at 30 and 36 months, and entomological inoculation rate (EIR) and vector density during the third year. Other entomological outcomes (changes in mosquito resting behaviour indoors and outdoors, species composition, host feeding, and mosquito ovary development and fecundity) were published elsewhere.[19] The frequency and intensity of phenotypic and genotypic resistance to various insecticides during the third year has also been reported elsewhere.[20]

Statistical analysis

The sample size was calculated for the 24-month endpoint. A total of 21 clusters per group and 45 households per cluster at each cross-sectional survey were determined to achieve 80% power to detect a 28% relative reduction in prevalence of malaria between each dual active ingredient LLIN group and the PY LLIN group, assuming a malaria prevalence of 40% in the reference group (PY LLIN) and a coefficient of variation of 21% as per our previous publication.[21] For the entomological endpoint, a mean EIR in the reference group of 1·76 infective mosquitoes per month per household was assumed with a between-cluster coefficient of variation of 0·4.[3] With eight households sampled in each cluster every quarter, or 32 collection house-nights per cluster per year, the study had 80% power to detect a relative reduction of 36% or more in monthly EIR between the intervention and reference groups. All sample size calculations allowed for a Bonferroni-corrected significance level of 1·67% (two-sided) to account for multiple comparisons.

Statistical analyses were done using Stata 15. The primary analyses of malaria infection prevalence and anaemia were based on intention to treat, defined as the entire population sampled. Secondary per-protocol analyses were conducted only for the main outcome, malaria infection prevalence, and only included children using the allocated study nets. For both intention-to-treat and per-protocol analyses, the repeated malaria infection prevalence measured at 30 and 36 months was analysed using mixed-effects logistic regression. The model included fixed effects for time (survey timepoint), study group, and the time by study group interaction. The cluster-level variables used in restricted randomisation (including the baseline measure of the primary outcome) were included as covariates; a random intercept was specified for clusters to account for intracluster correlation and robust standard errors were added to account for possible mis-specification of the variance structure of the errors.[22] Results from the analyses of the 30 and 36 months timepoints were also plotted alongside results from the primary trial endpoint at 24 months to visualise trends. The treatment effects for both intention-to-treat and per-protocol analyses were estimated using adjusted least-squares mean differences with 95% CIs obtained from the respective models. Adjusted risk ratios for malaria prevalence in the intention-to-treat population were also estimated.

Vector density and EIR in the third year were analysed by intention to treat and compared between study groups using a mixed-effects negative binomial regression model. A mixed-effects logistic regression model was used to analyse the sporozoite rates. Both models

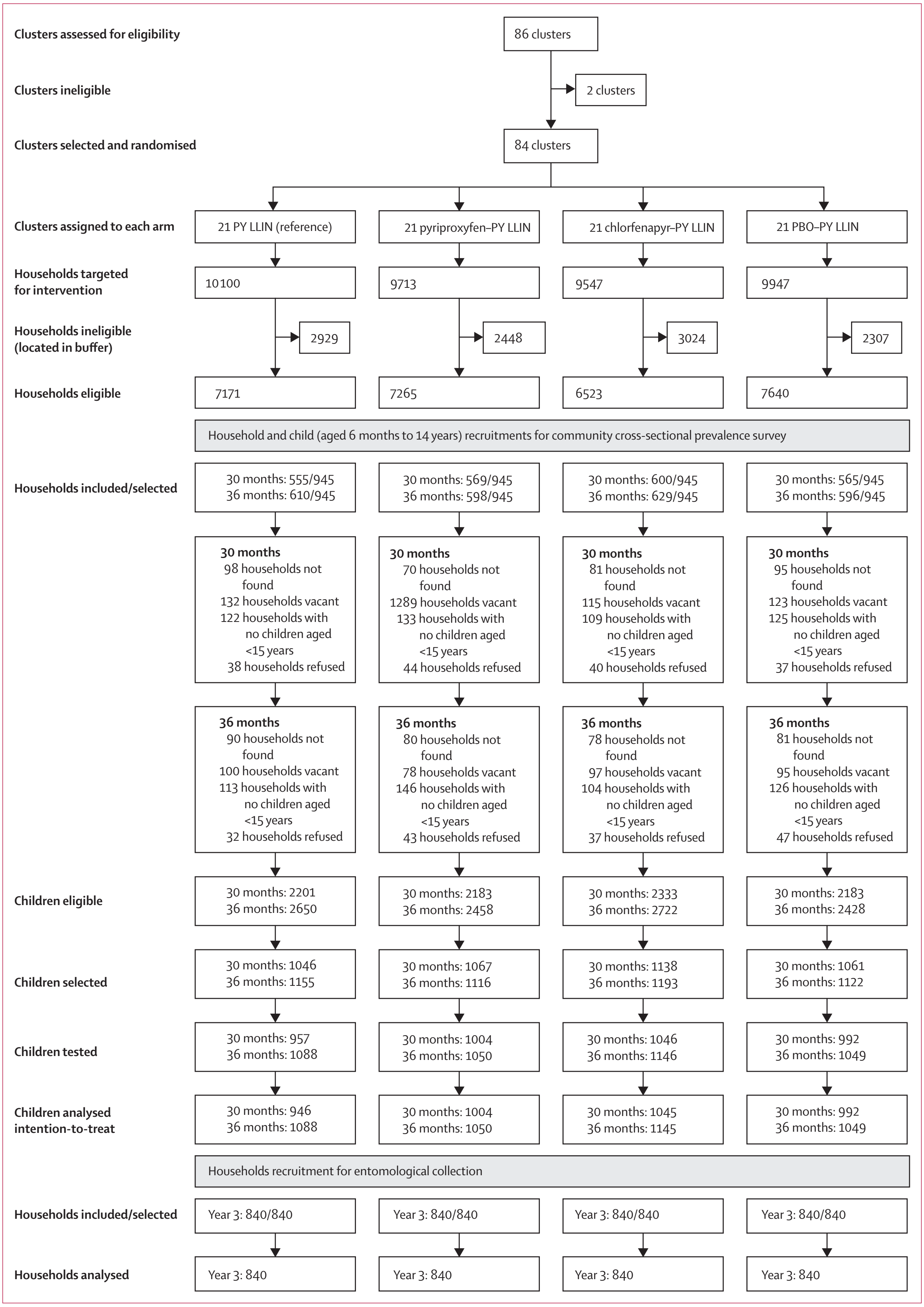

***Figure 1:* Trial profile**
PY=pyrethroid. LLIN=long-lasting insecticidal net. PBO=piperonyl butoxide.

	PY LLINs (reference)	Pyriproxyfen–PY LLINs	Chlorfenapyr–PY LLINs	PBO–PY LLINs
**Study cluster characteristics**				
Population (whole study area)	61 183	57 567	60 115	57 631
Population (core area)	43 877	43 266	41 748	45 020
**Household and children characteristics baseline cross-sectional survey (September, 2018)**				
SES (poorest households)	114/394 (28·9%)	141/390 (36·1%)	131/426 (30·7%)	122/333 (36·6%)
LLIN use in all age groups	2957/4962 (59·6%)	2813/4520 (62·2%)	2849/4803 (59·3%)	2695/4369 (61·7%)
LLIN use in selected children	831/1295 (63·2%)	839/1290 (64·9%)	781/1249 (62·5%)	751/1185 (63·4%)
Malaria infection prevalence	2957/4962 (59·6%)	516/1118 (46·2%)	469/1099 (42·7%)	444/1056 (42·0%)
Anaemia prevalence in children aged under 5 years (<8 g/dL)	28/453 (6·1%)	28/500 (5·2%)	28/507 (5·5%)	20/472 (4·2%)
Median (IQR) age of selected children, years	6 (3–10), n=1295	6 (3–10), n=1290	6 (3–9), n=1249	6 (3–10), n=1185
**Entomological characteristics (September–December, 2018)**				
Mean (95% CI) indoor vector per house per night	5·9 (0·7–11·1), n=335	4·2 (0·8–7·6), n=334	2·8 (0·0–6·0), n=334	1·9 (0·4–3·4), n=337
Sporozoite rate	30/680 (4·4%)	19/570 (3·3%)	7/318 (2·2%)	11/367 (3·0%)
EIR (95% CI) per household per night	0·35 (0·02–0·68), n=331	0·11 (0·01–0·21), n=328	0·04 (0·00–0·08), n=332	0·07 (0·01–0·13), n=326
Proportion of *A funestus*	1869/1982 (94·3%)	1331/1401 (95·0%)	904/948 (95·4%)	596/642 (92·8%)

Data are n or n/N (%), unless otherwise specified. PY=pyrethroid. LLIN=long-lasting insecticidal net. PBO=piperonyl butoxide. SES=socioeconomic status. EIR=entomological inoculation rate.

Table 1: Baseline characteristics

included fixed effects for study group and cluster-level variables used in restricted randomisation with collection round and cluster as random effects. Secondary analysis across the 3 years of collection was performed to assess trends in vector density at each survey period between the intervention groups. In this analysis, the model included fixed effects for year, study group, and the interaction year by study group, and adjusted for the cluster-level variables used in restricted randomisation, with collection round and cluster as random effects. Robust standard errors were added to account for possible mis-specification of the variance structure of the errors.

See Online for appendix

This trial is registered with ClinicalTrials.gov (NCT03554616) and is now completed.

Role of the funding source

The funders of the trial had no role in study design, data collection, data interpretation, or writing of the report, or in the decision to submit for publication.

Results

The study area included 39 307 households (132–2390 households per cluster) and 236 496 people, evenly distributed between the four groups (figure 1). Of these, 23 911 households (60·8%) were eligible for cross-sectional surveys of malaria prevalence. For each of the two survey rounds in the third year, 7560 households were randomly selected. Across these surveys, 4722 (62·5%) households consented, 978 (12·9%) had no children under 15 years of age, 868 (11·5%) were absent, 199 (2·6%) refused, and 318 (4·2%) were not located (figure 1). In the households that consented, 8898 children were selected, 8332 came to be tested, and 8329 were included in the intention-to-treat analysis (figure 1).

During the baseline survey, which was conducted in 2018, all children, household, and cluster characteristics were similar between the four trial groups (table 1)[4] and at 30 and 36 months post intervention (appendix p 3). Overall usage of LLINs (study nets and other nets) was 2987 (69·3%) of 4310 at 30 months, and increased slightly after the school net distribution to 3311 (72·2%) of 4587 at 36 months. Usage was similar between groups (appendix p 4), with the study LLIN usage being 1325 (30·7%) of 4310 at 30 months and 1023 (22·3%) of 4587 at 36 months. Generally, study net usage was low, with PBO–PY LLIN usage the lowest followed by that of pyriproxyfen–PY LLINs. At 30 months, 2816 (46·0%) of the 6124 nets owned in the study area were study nets, 2438 (39·8%) were various brands of standard PY LLIN, and 723 (11·8%) were PBO–PY LLINs distributed by government programmes. At 36 months, the proportion of study nets owned declined to 2222 (31·2%) of 7129; the remaining were PBO–PY LLINs (2568 [36·0%] of 7129 from the school net distribution in October, 2021) and standard PY LLINs (2162 [30·3%] of 7125 distributed from antenatal clinics; appendix pp 5–8). Chemical analysis showed that at 36 months, partner active ingredient retention was 1·5 g/kg (28%) of an initial 5·4 g/kg for pyriproxyfen, 0·4 (8%) of 5·0 for chlorfenapyr, and 0·7 (7%) of 9·6 for PBO, with PY retention lowest in standard PY LLINs (0·8 [17%] of 4·7) and highest in pyriproxyfen–PY LLINs (3·3 [62%] of 5·3; appendix p 9).

In the intention-to-treat analysis, at 36 months, malaria infection prevalence in children was 407 (37·4%) of 1088 in the standard PY LLIN group versus 261 (22·8%) of 1145 in the chlorfenapyr–PY LLIN group (OR 0·57, 95% CI 0·38–0·86; p=0·0069), 338 (32·3%) of 1048 in the PBO–PY LLIN group (0·95, 0·64–1·42; p=0·80), and

	Intention to treat					Per protocol				
	n/N	%	OR	95% CI	p value	n/N	%	OR	95% CI	p value
**30 months**										
PY LLINs	507/956	53·0%	1 (ref)	··	··	195/388	50·3%	1 (ref)	··	··
Chlorfenapyr–PY LLINs	436/1045	41·7%	0·69	0·46–1·03	0·069	123/360	34·2%	0·52	0·33–0·82	0·0052
PBO–PY LLINs	488/992	49·2%	0·99	0·66–1·48	0·97	94/186	50·5%	1·05	0·64–1·70	0·87
Pyriproxyfen–PY LLINs	426/1004	42·4%	0·73	0·49–1·09	0·12	110/306	35·9%	0·54	0·33–0·85	0·0086
**36 months**										
PY LLINs	407/1088	37·4%	1 (ref)	··	··	110/335	32·8%	1 (ref)	··	··
Chlorfenapyr–PY LLINs	261/1145	22·8%	0·57	0·38–0·86	0·0069	60/318	18·9%	0·50	0·30–0·83	0·0068
PBO–PY LLINs	338/1049	32·2%	0·95	0·64–1·42	0·80	33/140	23·6%	0·70	0·40–1·25	0·23
Pyriproxyfen–PY LLINs	302/1050	28·8%	0·82	0·55–1·23	0·34	53/192	27·6%	0·78	0·45–1·32	0·35

Each intervention arm is compared with the PY LLIN arm for the same timepoint. Rate ratios are adjusted for baseline cluster-level variables used in restricted randomisation. A p value <0·017 was considered statistically significant after Bonferroni correction. OR=odds ratio. PY=pyrethroid. LLIN=long-lasting insecticidal net. PBO=piperonyl butoxide.

Table 2: **Malaria infection prevalence in children aged 6 months to 14 years at 30 and 36 months post intervention in intention-to-treat and per-protocol analyses**

302 (28·8%) of 1050 in the pyriproxyfen–PY LLIN group (0·82, 0·55–1·23; p=0·34; table 2). At 30 months, when compared with the standard PY LLIN group there was weak evidence of a difference in malaria infection prevalence in the three intervention groups (table 2). Risk ratios for the intention-to-treat analysis are reported in the appendix (p 10). The per-protocol analysis showed a similar effect to the intention-to-treat analysis except for at 30 months, where children sleeping under a pyriproxyfen–PY LLIN (0·54, 0·33–0·85; p=0·0086) or under a chlorfenapyr–PY LLIN (0·52, 0·33–0·82; p=0·0052) were more protected than those sleeping under a standard PY LLIN. None of the participants reported side-effects at 30 or 36 months.

Comparing the parasite prevalence ORs in all five surveys post intervention, households provided with chlorfenapyr–PY LLINs had the lowest parasite prevalence compared with households who received standard PY LLINs, and this effect was sustained in all three years (figure 2A). For PBO–PY LLINs the effect was observed up to 18 months, compared with only 12 months for pyriproxyfen–PY LLINs (figure 2A). Relative to the standard PY LLIN group, there was no significant difference observed in the prevalence of anaemia in any of the intervention groups at any of the timepoints (appendix p 11).

A total of 44 007 mosquitoes were collected during the third year of follow-up across the study groups, of which 8260 (18·8%) were female anophelines, mainly *A funestus* ss and *Anopheles arabiensis*. During the third year, vector density was 2·72 in the standard PY LLIN group compared with 2·01 in the chlorfenapyr–PY LLIN group (density ratio 0·46, 95% CI 0·28–0·74; p=0·0017), 3·01 in the pyriproxyfen–PY LLIN group (0·86, 0·53–1·39; p=0·53), and 2·10 in the PBO–PY LLIN group (0·64, 0·40–1·03; p=0·047; table 3). EIR was generally lower for all the intervention groups versus the standard PY LLIN group, with only the chlorfenapyr–PY LLIN group showing evidence of a difference. Comparing the vector population density each post-intervention year, *Anopheles* densities were significantly lower in the chlorfenapyr–PY LLIN group over the 3 years compared with the standard PY LLIN group (figure 2B). The effect of PBO–PY LLINs was strongest during the first year of follow-up and only borderline reductions were observed in the following 2 years (figure 2B).

Discussion

This study generated additional evidence on the performances of three classes of dual active ingredient LLINs over their full 3-year lifespan compared with standard PY LLINs. We found that chlorfenapyr–PY LLINs provided significantly better protection against malaria infection prevalence during the third year of follow-up compared with standard PY LLINs. Children under 15 years of age in the chlorfenapyr–PY LLIN group had 43% reduced odds of malaria prevalence relative to those from the clusters deployed with standard PY LLINs at 36 months post intervention. Similar effects were observed against entomological indices, whereby there was a significant 54% decline in vector density and 68% reduction in EIR in the chlorfenapyr–PY LLIN group compared with the standard PY LLIN group. As reported in our previous Article on the first 2 years of the trial,[4] and confirmed by the third-year results presented in this paper, there was limited additional protection afforded by PBO–PY LLINs or pyriproxyfen–PY LLINs compared with standard PY LLINs against malaria infection prevalence, transmission (measured by EIR), or vector density in the third year post LLIN distribution.

We observed continued superior protection in the chlorfenapyr–PY LLIN group against malaria infection prevalence, transmission, and vector density compared with the standard PY LLIN group after 36 months of

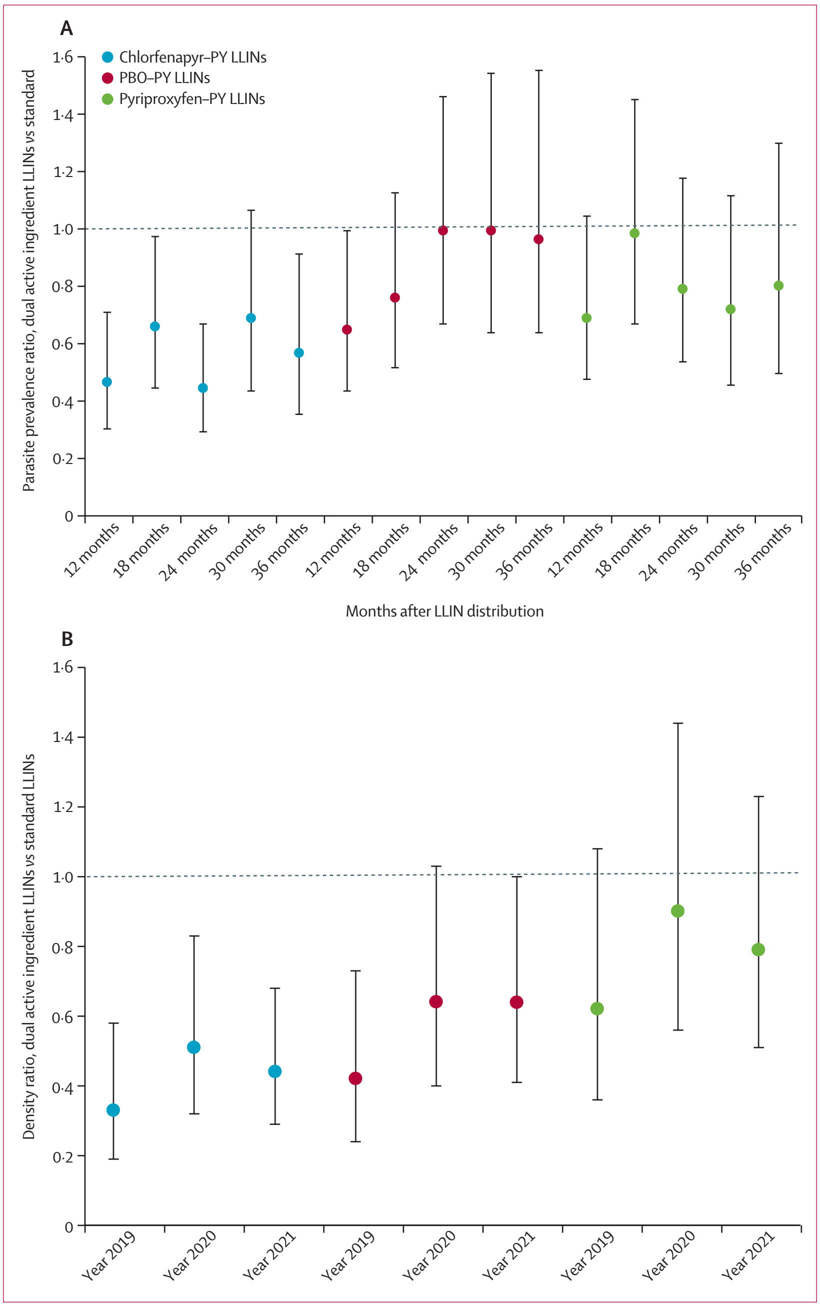

Figure 2: **Trends in (A) parasite prevalence and (B) vector density of dual active ingredient LLINs versus standard PY LLINs**

Observed at 12, 18, 24, 30, and 36 months post LLIN distribution. Parasite prevalence is presented as odds ratios as per the intention-to-treat analysis. Error bars show 95% CIs. Dashed lines show the reference value. LLIN=long-lasting insecticidal net. PBO=piperonyl butoxide. PY=pyrethroid.

follow-up, when usage of the chlorfenapyr–PY LLINs was below 31% and only 8% of chlorfenapyr content remained. Our findings are supported by the recent Benin randomised controlled trial,[6] which found that over 2 years of follow-up there was strong evidence of a reduction in malaria case incidence by 46% in the chlorfenapyr–PY LLIN group relative to the standard PY LLIN group. The significant reduction in EIR over the 3 years suggests a sustained level of community protection, which is further supported by the fact that the reduction in malaria prevalence was only slightly stronger among children who actually reported using a chlorfenapyr–PY LLIN (per protocol). We reported previously that, after 2 years, chlorfenapyr–PY LLINs were already the most cost-effective LLIN from a societal and household perspective, relative to standard PY LLINs, PBO–PY LLINs, and pyriproxyfen–PY LLINs. The sustained effectiveness of chlorfenapyr–PY LLINs over the 3 years is thus likely to increase estimates of cost saving from the donor and public provider perspectives.[4] However, our study is likely to have underestimated the true impact of chlorfenapyr–PY LLINs due to low usage; therefore, strategies to keep net usage high need to be devised to maximise the benefit of these new LLIN tools for malaria control. The third-year results of the Benin randomised controlled trial, expected in late 2023, will also be important in determining whether the effect is sustained over 3 years in a different trial setting where vector composition is different and vector density and malaria transmission are much higher than in Tanzania, especially in light of reports of reduced susceptibility to chlorfenapyr–PY in *A gambiae* ss in this setting.[23]

In this paper, we report that the effectiveness of PBO–PY LLINs lasted for 18 months, and little or no additional protective efficacy against malaria prevalence or entomological outcomes was observed in the third year compared with standard PY LLINs. In previous randomised controlled trials, conducted in Uganda[24] and in a different region in Tanzania (Muleba), PBO–PY LLINs showed protective efficacy for up to 21–25 months of use in Uganda[25] and after 3 years in Tanzania.[15] Notable differences between our present study and the other two are the species composition and variations in insecticide resistance in mosquitoes vectors, as well as differences in PBO–PY LLIN usage and potential differences in human behaviour. In our current trial, *A funestus* is the main vector in the study area,[19] as opposed to *A gambiae* ss in the trials in Uganda[24] and Muleba, Tanzania.[15] Another possible explanation for the differential effects of PBO–PY LLINs between trials could be differences in chemical specificity and textile durability of the nets in the present study, with textile durability likely to be the main driver of the fast decline in usage of this type of net in the community. In the Uganda trial, the PY content of PBO–PY LLINs was relatively stable at 12 and 25 months of evaluation, but the PBO content declined by 55%,[26] with a similar brand used in our current trial. After 24 months of follow-up, 29% of PBO remained, and after 36 months, only 7% was left in the study nets from the present randomised controlled trial. In the present trial, usage of PBO–PY LLINs dropped much more quickly during the first year, from 74% at 3 months post intervention to 59% at 12 months, 30% at 24 months, and 11% at 36 months; in

	Households analysed	Vector density				SR					EIR			
		Mean density per household per night	DR	95% CI	p value	*Anopheles* tested	SR %	OR	95% CI	p value	EIR per household per night	DR*	95% CI	p value
PY LLINs	840	2·72	1 (ref)	..	..	1717	1·86%	1 (ref)	..	..	0·04	1 (ref)	..	..
Chlorfenapyr–PY LLINs	840	2·01	0·46	0·28–0·74	0·0017	1233	0·97%	0·61	0·27–1·40	0·24	0·02	0·32	0·15–0·71	0·0046
PBO–PY LLINs	840	2·10	0·64	0·40–1·03	0·047	1304	1·69%	0·92	0·46–1·85	0·82	0·03	0·64	0·26–1·61	0·34
Pyriproxyfen–PY LLINs	840	3·01	0·86	0·53–1·39	0·53	1732	0·81%	0·46	0·21–1·00	0·051	0·02	0·47	0·19–1·21	0·12

DR for vector density and EIR and OR for SRs are adjusted for baseline cluster-level variables used in restricted randomisation. A p value <0·017 was considered statistically significant after Bonferroni correction. SR=sporozoite rate. EIR=entomological inoculation rate. DR=density ratio. OR=odds ratio. PY=pyrethroid. LLIN=long-lasting insecticidal net. PBO=piperonyl butoxide. *EIR DRs are weighted to account for the proportion of mosquitoes sampled to be tested for sporozoites.

Table 3: **Entomological outcomes during the third year post distribution in intention-to-treat analysis**

the other Tanzania study, usage was 76% at 4 months and 46% at 24 months, and in the Uganda study, overall net usage was 80% at 12 months and 60% at 25 months. In our study, the community had access to other types of new nets; therefore, torn nets were easily replaced. This is in contrast to the previous Tanzania trial,[3] in which communities lacked access to other nets and as a result households retained their PBO–PY LLINs for longer.[27]

Although we observed little to no reduction for any of the outcomes in the pyriproxyfen–PY LLIN group during the second year of the trial, or for entomological outcomes in the third year, there was some evidence of a reduction in malaria infection prevalence among children using pyriproxyfen–PY LLINs compared with standard PY LLINs during the 30-month follow-up. A higher concentration of alpha-cypermethrin remaining in the pyriproxyfen–PY LLINs (3·3 g/kg) compared with standard PY LLINs (0·8 g/kg) in the third year could explain these results; however, this should be confirmed using standard WHO bioefficacy testing. Other trials have reported limited protection of pyriproxyfen–PY LLINs relative to standard PY LLINs on malaria outcomes. For example, in a randomised controlled trial in Burkina Faso, over 18 months of follow-up there was a low (12%) reduction in malaria case incidence among children using pyriproxyfen–PY LLINs compared with those using standard PY LLINs.[5] In the Benin trial, the pyriproxyfen–PY LLINs did not provide additional protection towards malaria infection prevalence;[5] however, these LLINs were effective in reducing indoor vector density by 22% and EIR by 51% compared with standard PY LLINs.[5] The variations in the epidemiological and entomological impact of these nets across the three trials could be partly explained by different net usage, time of follow-up, and also resistance intensity to pyriproxyfen or the partner PY used on the LLIN.[5,6,20,28]

This study has limitations, including the low study net usage during the third year of follow-up, which might explain the lack of impact offered by each of the three intervention nets at this timepoint. The study was subject to some methodological limitations, including potential measurement bias in intention-to-treat estimates and selection bias in per-protocol estimates. The distribution of PBO–PY LLINs was in October, 2021, before the end of the third year of trial follow-up; although based on secondary analysis, the findings showed that ownership and usage of non-study nets was balanced across the groups, and thus less likely to introduce bias on the study outcomes. However, the addition of PBO–PY LLINs might have differential impact in the four groups: first, because PBO is known to synergise the efficacy of pyriproxyfen, whereas it could have a negative antagonistic impact when combined with a pro-insecticide such as chlorfenapyr;[29] and second, because of the differences in malaria transmission (EIR) between the four groups.[28,30]

Based on the present study and the study in Benin,[6] the WHO Vector Control Advisory Group concluded that chlorfenapyr–PY LLINs were more effective than standard PY LLINs in areas with highly PY-resistant malaria vectors,[7] and WHO has released a policy recommendation of these nets for public implementation against malaria in areas with PY resistance.[9]

This study demonstrates the sustained effectiveness of chlorfenapyr–PY LLINs in an area of intense insecticide resistance, despite relatively low levels of coverage, which adds to the evidence base in support of these new nets as a highly promising malaria control tool. Appropriate net replacement strategies and social behaviour communication are needed to keep LLIN usage high and maximise impact. Moreover, insecticide resistance plans should incorporate strategies to preserve chlorfenapyr efficacy to prevent or delay resistance development in malaria vectors. Importantly, additional combinations of vector control products should still be considered an integral part of tailored vector control strategies, especially as evidence of chlorfenapyr resistance has been reported in wild populations. This study highlights the urgent need for improved durability of dual active ingredient LLINs, alongside strategies to improve community awareness and usage of these nets, to optimise the effectiveness of these new nets for sustainable malaria control.

Contributors
NP, MAK, MR, FWM, and IK conceived the idea and designed the study. NP, MAK, FWM, MR, AM, and IK secured the funding for the study. NP, MAK, and MT developed the data analysis plan. FWM and AM supported the trial implementation by engaging both local and national stakeholders. JFM, NSM, EL, EM, and TA led in the field data collection and NP provided overall supervision and management of the project. JFM, NSM, MAK, MT, and NP were involved in the statistical analysis of the data collected. EM assisted in the data management. NSM and JFM wrote the first draft of the manuscript. NP, MAK, and LAM interpreted the data and critically reviewed the manuscript. All authors contributed to the revision and editing of the manuscript. JFM, NSM, and NP had full access to all the data in the study and had final responsibility for the decision to submit for publication. All authors reviewed, read, and approved the final version of the manuscript.

Declaration of interests
We declare no competing interests.

Data sharing
De-identified data and a data dictionary will be made available upon publication following reasonable request to the corresponding authors (Department of Parasitology, National Institute for Medical Research, Mwanza Medical Research Centre, Mwanza, Tanzania; Department of Disease Control, London School of Hygiene & Tropical Medicine, London, UK). The trial protocol has been published previously.[18]

Acknowledgments
The trial was funded by the Joint Global Health Trials (MR/R006040/1), Department for International Development, UK Medical Research Council, Wellcome Trust, Department of Health and Social Care, and Bill & Melinda Gates Foundation via the Innovative Vector Control Consortium. We thank colleagues and staff at the Kilimanjaro Christian Medical University College and at the National Institute of Medical Research Mwanza who were involved in the project. We are grateful for the continuous support provided by the Tanzanian National Malaria Control Program, the President's Office—Regional Administration and Local Government, and the Regional Medical office in Mwanza. We are particularly indebted to the Misungwi District Health Office and the community health workers for facilitating and supervising the different project activities. We thank USAID/President's Malaria Initiative for funding TA to carry out the behaviour change communications during the LLIN distribution campaign.

References
1 WHO. World malaria report. 2021. https://www.who.int/teams/global-malaria-programme/reports/world-malaria-report-2021 (accessed Dec 13, 2022).

2 Hemingway J, Ranson H, Magill A, et al. Averting a malaria disaster: will insecticide resistance derail malaria control? *Lancet* 2016; **387:** 1785–88.

3 Protopopoff N, Mosha JF, Lukole E, et al. Effectiveness of a long-lasting piperonyl butoxide-treated insecticidal net and indoor residual spray interventions, separately and together, against malaria transmitted by pyrethroid-resistant mosquitoes: a cluster, randomised controlled, two-by-two factorial design trial. *Lancet* 2018; **391:** 1577–88.

4 Mosha JF, Kulkarni MA, Lukole E, et al. Effectiveness and cost-effectiveness against malaria of three types of dual-active-ingredient long-lasting insecticidal nets (LLINs) compared with pyrethroid-only LLINs in Tanzania: a four-arm, cluster-randomised trial. *Lancet* 2022; **399:** 1227–41.

5 Tiono AB, Ouedraogo A, Ouattara D, et al. Efficacy of Olyset Duo, a bednet containing pyriproxyfen and permethrin, versus a permethrin-only net against clinical malaria in an area with highly pyrethroid-resistant vectors in rural Burkina Faso: a cluster-randomised controlled trial. *Lancet* 2018; **392:** 569–80.

6 Accrombessi M, Cook J, Dangbenon E, et al. Efficacy of pyriproxyfen-pyrethroid long-lasting insecticidal nets (LLINs) and chlorfenapyr-pyrethroid LLINs compared with pyrethroid-only LLINs for malaria control in Benin: a cluster-randomised, superiority trial. *Lancet* 2023; **401:** 435–46.

7 WHO Vector Control Advisory Group. Seventeenth meeting of the WHO Vector Control Advisory Group. 2022. https://www.who.int/publications/i/item/9789240066021 (accessed Dec 13, 2022).

8 WHO Vector Control Advisory Group. Fifteenth meeting of the WHO Vector Control Advisory Group. 2021. https://www.who.int/publications/i/item/9789240040854 (accessed Dec 13, 2022).

9 WHO. WHO publishes recommendations on two new types of insecticide-treated nets. 2023. https://www.who.int/news/item/14-03-2023-who-publishes-recommendations-on-two-new-types-of-insecticide-treated-nets (accessed June 13, 2023).

10 WHO. Guidelines for laboratory and field-testing of long-lasting insecticidal nets. 2013. https://www.who.int/publications/i/item/9789241505277 (accessed Dec 13, 2022).

11 Mansiangi P, Umesumbu S, Etewa I, et al. Comparing the durability of the long-lasting insecticidal nets DawaPlus® 2.0 and DuraNet© in northwest Democratic Republic of Congo. *Malar J* 2020; **19:** 189.

12 Kilian A, Koenker H, Obi E, Selby RA, Fotheringham M, Lynch M. Field durability of the same type of long-lasting insecticidal net varies between regions in Nigeria due to differences in household behaviour and living conditions. *Malar J* 2015; **14:** 123.

13 Massue DJ, Moore SJ, Mageni ZD, et al. Durability of Olyset campaign nets distributed between 2009 and 2011 in eight districts of Tanzania. *Malar J* 2016; **15:** 176.

14 Lukole E, Cook J, Mosha JF, et al. Protective efficacy of holed and aging PBO-pyrethroid synergist-treated nets on malaria infection prevalence in north-western Tanzania. *PLoS Glob Public Health* 2022; **2:** e0000453.

15 Protopopoff N, Mosha JF, Messenger LA, et al. Effectiveness of three-year old piperonyl butoxide and pyrethroid-treated long-lasting insecticidal nets (LLINs) versus pyrethroid-only LLINs against malaria infection: results of a cluster randomised trial in Tanzania. *medRxiv* 2022; published online July 7. https://doi.org/10.1101/2022.07.06.22277292 (preprint).

16 N'Guessan R, Odjo A, Ngufor C, Malone D, Rowland M. A chlorfenapyr mixture net Interceptor® G2 shows high efficacy and wash durability against resistant mosquitoes in West Africa. *PLoS One* 2016; **11:** e0165925.

17 Gichuki PM, Kamau L, Njagi K, et al. Bioefficacy and durability of Olyset® Plus, a permethrin and piperonyl butoxide-treated insecticidal net in a 3-year long trial in Kenya. *Infect Dis Poverty* 2021; **10:** 135.

18 Mosha JF, Kulkarni MA, Messenger LA, et al. Protocol for a four parallel-arm, single-blind, cluster-randomised trial to assess the effectiveness of three types of dual active ingredient treated nets compared to pyrethroid-only long-lasting insecticidal nets to prevent malaria transmitted by pyrethroid insecticide-resistant vector mosquitoes in Tanzania. *BMJ Open* 2021; **11:** e046664.

19 Matowo NS, Martin J, Kulkarni MA, et al. An increasing role of pyrethroid-resistant *Anopheles funestus* in malaria transmission in the Lake Zone, Tanzania. *Sci Rep* 2021; **11:** 13457.

20 Messenger LA, Matowo NS, Cross CL, et al. Effects of next-generation, dual-active-ingredient, long-lasting insecticidal net deployment on insecticide resistance in malaria vectors in Tanzania: an analysis of a 3-year, cluster-randomised controlled trial. *Lancet Planet Health* 2023; **7:** E673–83.

21 West PA, Protopopoff N, Wright A, et al. Indoor residual spraying in combination with insecticide-treated nets compared to insecticide-treated nets alone for protection against malaria: a cluster randomised trial in Tanzania. *PLoS Med* 2014; **11:** e1001630.

22 Hooper R, Teerenstra S, de Hoop E, Eldridge S. Sample size calculation for stepped wedge and other longitudinal cluster randomised trials. *Stat Med* 2016; **35:** 4718–28.

23 Tchouakui M, Assatse T, Tazokong HR, et al. Detection of a reduced susceptibility to chlorfenapyr in the malaria vector *Anopheles gambiae* contrasts with full susceptibility in *Anopheles funestus* across Africa. *Sci Rep* 2023; **13:** 2363.

24 Maiteki-Sebuguzi C, Gonahasa S, Kamya MR, et al. Effect of long-lasting insecticidal nets with and without piperonyl butoxide on malaria indicators in Uganda (LLINEUP): final results of a cluster-randomised trial embedded in a national distribution campaign. *Lancet Infect Dis* 2022; **23:** 247–58.

25 Gleave K, Lissenden N, Chaplin M, Choi L, Ranson H. Piperonyl butoxide (PBO) combined with pyrethroids in insecticide-treated nets to prevent malaria in Africa. *Cochrane Database Syst Rev* 2021; **5:** CD012776.

26 Mechan F, Katureebe A, Tuhaise V, et al. LLIN Evaluation in Uganda Project (LLINEUP): the fabric integrity, chemical content and bioefficacy of long-lasting insecticidal nets treated with and without piperonyl butoxide across two years of operational use in Uganda. *Curr Res Parasitol Vector Borne Dis* 2022; **2:** 100092.

27 Martin JL, Mosha FW, Lukole E, et al. Personal protection with PBO-pyrethroid synergist-treated nets after 2 years of household use against pyrethroid-resistant *Anopheles* in Tanzania. *Parasit Vectors* 2021; **14:** 150.

28 Matowo NS, Kulkarni MA, Messenger LA, et al. Differential impact of dual-active ingredient long-lasting insecticidal nets on primary malaria vectors: a secondary analysis of a 3-year, single-blind, cluster-randomised controlled trial in rural Tanzania. *Lancet Planet Health* 2023; **7:** e370–80.

29 Syme T, Gbegbo M, Obuobi D, et al. Pyrethroid-piperonyl butoxide (PBO) nets reduce the efficacy of indoor residual spraying with pirimiphos-methyl against pyrethroid-resistant malaria vectors. *Sci Rep* 2022; **12:** 6857.

30 Sherrard-Smith E, Winskill P, Hamlet A, et al. Optimising the deployment of vector control tools against malaria: a data-informed modelling study. *Lancet Planet Health* 2022; **6:** e100–09.
